# Targeted Delivery Inside the Cells Directly Visualized with Förster Resonance Energy Transfer (FRET)

**DOI:** 10.3390/polym17060790

**Published:** 2025-03-16

**Authors:** Igor D. Zlotnikov, Natalya G. Belogurova, Elena V. Kudryashova

**Affiliations:** Faculty of Chemistry, Lomonosov Moscow State University, Leninskie Gory, 1/3, 119991 Moscow, Russia; zlotnikovid@my.msu.ru (I.D.Z.);

**Keywords:** curcumin, umbelliferones, polymeric micelles, FRET, RFP, bacteria cells targeting

## Abstract

We established a real-time Förster resonance energy transfer (FRET) based assay to evaluate targeted drug delivery using polymeric micelles. Red fluorescent protein (RFP)-expressing *E. coli* cells were used as a test system to monitor the delivery of drug-fluorophore such as curcumin and umbelliferones (MUmb and AMC) encapsulated in the polymeric micellar formulations. The efficiency of the drug delivery was quantified using the FRET efficiency, measured as the degree of energy transfer from the drug to the RFP. FRET efficiency directly provides the determination of the delivery efficacy, offering a versatile platform adaptable to various drugs and cell types. We used polymer micelles as a carrier for targeted delivery of fluorescent drugs to bacterial cells expressing RFP. The physicochemical characterization of the interaction between the drugs and the micelles including spectral properties, and the solubility and binding constants, were determined. We revealed a stronger affinity of MUmb for heparin-based micelles (*K*_d_~10^−5^ M) compared to chitosan-based micelles (*K*_d_~10^−4^ M), underscoring the influence of polymer composition on drug loading efficiency. For micelles containing MUmb, a FRET efficiency significantly exceeds (by three times) the efficiency for non-micellar MUmb, which have minimal penetration into bacterial cells. The most noticeable effect was observed with the use of the micellar curcumin providing pronounced activation of the RPF fluorescence signal, due to the interaction with curcumins (fluorophore-donor). Curcumin delivery using Chit5-OA micelle resulted in a 115% increase in RFP fluorescence intensity, and Hep-LA showed a significant seven-fold increase. These results highlight the significant effect of micellar composition on the effectiveness of drug delivery. In addition, we have developed a visual platform designed to evaluate the effectiveness of a pharmaceutical product through the visualization of the fluorescence of a bacterial culture on a Petri dish. This method allows us to quickly and accurately assess the penetration of a drug into bacteria, or those located inside other cells, such as macrophages, where the intercellular latent forms of the infection are located. Micellar formulations show enhanced antibacterial activity compared to free drugs, and formulations with Hep-OA micelles demonstrate the most significant reduction in *E. coli* viability. Synergistic effects were observed when combining curcumin and MUmb with moxifloxacin, resulting in a remarkable 40–50% increase in efficacy. The presented approach, based on the FRET test system with RFP expressed in the bacterial cells, establishes a powerful platform for development and optimizing targeted drug delivery systems.

## 1. Introduction

Bacterial infections constitute a significant public health challenge that necessitates the creation of novel and efficient therapeutic approaches [1,2,3,4,5,6]. Modern antibacterial drugs of the third and fourth generation effectively destroy pathogenic bacteria in in vitro conditions. However, in in vivo conditions, they do not penetrate into the localization sites of the pathogen well, this primarily concerns macrophages, where latent forms of pathogens, in particular *Mycobacteria*, *Brucellae*, *C. Pneumoniae* are localized. The low efficiency of the drug delivery forces people to take high doses of antibacterial drugs, sometimes for many months, causing significant side effects. The limited efficacy of current antimicrobial therapies stems from several key factors. Poor antibiotic penetration into sites of latent infection and the action of bacterial efflux pumps—proteins that actively remove drugs from cells—are major contributors to treatment failure [7,8,9,10,11,12]. Strategies to increase drug exposure at the site of infection, even at the same or lower dosages, and to enhance targeted drug delivery, offer significant potential to improve both the effectiveness and safety of infectious disease treatments [13].

While extensive research over the past decade has investigated drug–bacteria interactions, a comprehensive understanding of the underlying molecular mechanisms remains elusive [5,14,15,16,17,18]. This study introduces a novel approach leveraging Förster resonance energy transfer (FRET), a powerful nanoscale technique for probing molecular interactions, to provide unprecedented insights into the complex interplay between bacterial cells and drug molecules [19,20,21,22]. This approach overcomes the limitations of previous studies by offering a quantitative, real-time assessment of these interactions.

Red fluorescent protein (RFP) is an intracellular fluorophore which is ideal for FRET-based studies due to the bright red fluorescence, thus minimizing background interference and enhancing signal specificity. This enhanced sensitivity could facilitate the precise study of protein–ligand interactions dynamics, intracellular signaling pathways, and other specific cellular processes, providing valuable instrument for various imaging applications [23,24,25,26], where RFP is mainly used for visualizing protein–protein interactions [27].

Here, we applied the bacteria expressing RFP as the FRET acceptor, and the drug molecule acting as the FRET fluorophore-donor. This configuration allows for the detection of a highly specific signal that indicates the interaction of a drug’s fluorescent molecule with bacterial cells expressing the fluorescent protein. The presence of a FRET signal would directly visualize and demonstrate the proximity of the drug to the bacteria, signifying successful drug delivery a drug distribution. We employed FRET technique to monitoring the interactions of bioactive molecules such as curcumin, 4-methylumbelliferone (MUmb), or 7-Amino-4-methylcoumarin (AMC) with RFP-containing bacterial cells. These compounds exhibit diverse modes of action. For instance, curcumin exhibits both anti-inflammatory and antimicrobial properties [17,28,29,30,31]. Preliminary studies suggest that umbelliferones, including MUmb, AMC, and their derivatives [32,33,34,35], may exhibit antibacterial, anti-inflammatory, and antioxidative properties. These compounds may serve as adjuvants for antibiotics in the treatment of a variety of bacterial infections, or they can be employed independently to prevent infections. The presented FRET pairs allow us to detect a highly specific signal corresponding to the interaction between the fluorescent drug molecule and bacterial cells expressing RFP.

However, non-optimal donor fluorophore biodistribution can limit this technique, resulting in an insufficient FRET signal. Targeted delivery of the fluorescent probe into the bacterial cell is therefore crucial. Employing a smart delivery system, such as polymeric micelles [18,36,37,38,39], is necessary to achieve effective intracellular probe localization and enhance FRET. To enhance selectivity towards bacterial cells, permeability, and retention, we employed our recently developed smart formulations that are based on trigger-responsive polymeric micelles derived from chitosan or heparin conjugated with fatty acid moieties [37,40,41,42]. Heparin’s antithrombotic properties are advantageous in inflammatory diseases often accompanied by thrombosis [43], whereas chitosan’s pH sensitivity [37,44,45], stemming from amino group protonation in the slightly acidic microenvironment surrounding bacteria, offers targeted delivery potential.

Polymeric micelles represent nanoscale entities that exhibit advantageous characteristics for drug delivery, including enhanced drug solubility and stability, prolonged circulation times, passive tumor targeting through the enhanced permeability and retention (EPR) effect [46,47,48], and the potential for stimuli-responsive drug release. Targeted delivery of antibiotics via micelles offers the potential to reduce systemic toxicity and enhance therapeutic efficacy [40,49,50,51,52,53,54].

This study aimed to develop a novel analytical approach based on the FRET technique for visualizing drug distribution dynamics, permeability into bacterial cells, and interactions with cellular components. Via FRET, we investigated the delivery of antibacterial drugs such as curcumin, AMC, or MUMb, and the effect of smart polymer micellar systems on the drug internalization. The findings are significant for elucidating bacterial cell–drug interactions and devising innovative approaches to combat bacterial infections (including intracellular). Our proposed FRET-based methodology offers a valuable tool for characterizing and optimizing delivery systems, including gene delivery. By labeling the nucleic acid with a suitable fluorophore, FRET with an intracellular acceptor can be used to monitor intracellular delivery. Changes in FRET efficiency directly report on the successful uptake and intracellular localization of the nucleic acid, providing quantitative data to assess the efficacy of different delivery vehicles and optimize their design for enhanced gene transfer.

## 2. Materials and Methods

### 2.1. Reagents

Chitosan lactate (5 kDa, Chit5), oleic acid (OA), lipoic acid (LA), spermine (sp), 1-ethyl-3-(3-dimethylaminopropyl) carbodiimide (EDC), N-hydroxysuccinimide (NHS), and 4-methylumbelliferone (MUmb) were purchased from Sigma-Aldrich (St. Louis, MO, USA). Curcumin and heparin (low-molecular-weight fractions, 12–14 kDa) were obtained from a commercial pharmacy with a certificate of quality. All other reagents, including salts, acids, and buffer components, were purchased from Reachim (Moscow, Russia).

### 2.2. Synthesis of Amphiphilic Polymers and Production of Polymeric Micelles

#### 2.2.1. Synthesis of Grafted Conjugates of Heparin and Chitosan

For Chit5-OA and Chit5-LA, a total of 20 mg of oleic and lipoic acids were dissolved in 5 mLof a mixture of CH_3_CN/PBS (4:1, *v*/*v*, pH 7.4). Then, 2.5 times the amount of EDC and 1.5 times the amount of NHS in DMSO were added to this mixture. The acid activation process was carried out for 20 min at a temperature of 30 °C. After that, pre-dissolved chitosan was added to the reaction mixture (50 mg in 10 mL of 1 mM HCl, followed by pH adjustment to 7). The mixture was incubated for 6 h at 50 °C. The reaction products were purified using centrifuge filters (3 kDa, 10,000× *g*, 3 times for 10 min), and then dialysis was performed against water for 12 h (with a cutoff of 6–8 kDa). The degree of chitosan modification was determined by spectrophotometric titration using 2,4,6-trinitrobenzoic sulfonic acid in a sodium-borate buffer (pH 9.2).

For Hep-OA, 125 mg of heparin was dissolved in 10 mL of PBS. Then, 2.5 times the amount of EDC and 1.3 times the amount of NHS relative to oleylamine in DMSO were added to the solution. This heparin activation process took place for 20 min at 30 °C. Next, pre-dissolved oleylamine (40 mg in 5 mL of CH_3_CN/PBS (4:1, *v*/*v*, pH 7.4)) was added and the mixture was incubated for 6 h at a temperature of 50 °C. The reaction mixtures were purified using centrifuge filters (10 kDa, 10,000× *g*, 3 times 10 min) and then subjected to dialysis against water for 12 h (with a cutoff of 12–14 kDa).

For Hep-LA, in order to synthesize heparin conjugate with lipoic acid (Hep-LA), heparin modified with spermine (with a 20% grafting degree) was first obtained, and then the residues of the lipoic acid were gradually added to the amino groups of spermine, similar to the protocol described above.

All samples were lyophilized at a temperature of −60 °C (Edwards 5, BOC Edwards, Crawley, UK).

#### 2.2.2. Production of Micelles

To obtain micelles, amphiphilic conjugates Chit5-LA, Chit5-OA, Hep-OA, and Hep-LA were dissolved in PBS (0.01 M, pH 7.4) at a concentration of 5 mg/mL. A preparation (MUmb or curcumin) in DMSO solvent (10 mg/mL) with a mass ratio of 0.1:1–10:1 (polymer/preparation) was added to these solutions. To form micelles and load the drug, the solutions were treated with ultrasound (at 50 °C, 10 min), and then extruded through a membrane with a pore size of 200 nm. To remove unrelated drugs, dialysis was performed for 6 h against PBS (with a 3 kDa cutoff).

### 2.3. Polymer and Micelle Characterization

Fourier-transform infrared (FTIR) spectroscopy was employed to characterize the chemical structure of the synthesized polymers and micelles. Spectra were recorded using a MICRAN-3 FTIR microscope (Simex, Novosibirsk, Russia) and a Bruker Tensor 27 spectrometer (Bruker, Ettlingen, Germany) equipped with a liquid-nitrogen-cooled mercury cadmium telluride (MCT) detector.

Further structural analysis was performed using ^1^H nuclear magnetic resonance (NMR) spectroscopy at 500 MHz using a Bruker Avance DRX 500 spectrometer (Bruker, Ettlingen, Germany). This provided detailed information on the proton environments within the polymer structures, confirming successful conjugation and grafting.

The degree of deacetylation in the chitosan (Chit5) was determined using circular dichroism (CD) spectroscopy on a Jasco J-815 CD spectrometer (JASCO, Tokyo, Japan). This analysis revealed a deacetylation degree of (92 ± 3)%.

The particle dimensions and surface charges were determined using the Malvern Zetasizer Nano ZS system (Malvern Instruments Ltd., Malvern, UK), which employs a HeNe laser (5 mW, 633 nm) as the light source. The measurements were conducted in a temperature-controlled cell at 25 degrees Celsius. The fluctuations in the light scattering intensity were analyzed using the autocorrelation function, which was obtained with the Malvern Correlator K7032-09 (Malvern Instruments Ltd., Malvern, UK).

Morphological characterization of the polymeric micelles, including size and shape analysis, was performed using atomic force microscopy (AFM) with an NT-MDT NTEGRA II AFM microscope (NT-MDT Spectrum Instruments, Moscow, Russia). AFM imaging allowed for a direct visual comparison of the morphology of the grafted chitosan-based micelles to that of unmodified chitosan, providing insights into the effects of modification on micelle formation and structure. This visual confirmation complemented the data obtained from other techniques, providing a comprehensive characterization of the synthesized materials.

### 2.4. Fluorescence and Absorption Spectra of Bioactive Molecules in the UV/Vis Range

The UV/Vis spectra of drug solutions (free form or micellar) in PBS (0.01 M, pH 7.4) were recorded on the AmerSham Biosciences UltraSpec 2100 pro device (Cambridge, UK). The fluorescence spectra of drug solutions (free form or micellar) in PBS (0.01 M, pH 7.4) were recorded using a Varian Cary Eclipse spectrofluorometer (Agilent Technologies, Santa Clara, CA, USA) at 37 °C.

### 2.5. Microbiological Studies

#### 2.5.1. Bacterial Culture and Preparation

*Escherichia coli* BL21(DE3) CGSC#12504 (F-, lon-11, Δ(ompT-nfrA)885, Δ(galM-ybhJ)884, λDE3 [lacI, lacUV5-T7 gene 1, ind1, sam7, nin5], Δ46, [mal+]K-12(λS), hsdS10) was used throughout this study. Overnight cultures (18–20 h, 37 °C, 120 rpm) were grown in Luria–Bertani (LB) broth (pH 7.2) to a cell density of approximately 4–7 × 10^8^ CFU/mL, as determined by both optical density (OD_600_) measurement and plate counting.

#### 2.5.2. Bacterial Transformation

Chemically competent *E. coli* cells were transformed with the pRed plasmid, encoding red fluorescent protein (RFP) cloned into the pQE30 vector (Qiagen Co., Hilden, Germany). The pRed plasmid was kindly provided by A.P. Savitsky (Institute of Biochemistry, Russian Academy of Science, Moscow, Russia). This transformation followed a standard protocol [55].

#### 2.5.3. Visualization of Petri Dishes with E. coli Expressing RFP and Interaction with Drugs Using the FRET Technique

##### RFP Expression Visualization

Following transformation and incubation (18 h at 37 °C, followed by a 2-day maturation period at 4 °C to enhance RFP fluorescence), *E. coli* colonies (10^7^ CFU/0.5 mL) were plated onto LB agar and imaged using a UVP BioSpectrum imaging system (UVP, Upland, CA, USA). Excitation and emission wavelengths were set at λ_ex, max_ = 480 nm and λem = 485–655 nm, respectively. This provided a macroscopic visual confirmation of RFP expression.

##### Visualization of Interactions of *E. coli* Expressing RFP with Drugs

*E. coli* expressing RFP (10^7^ CFU/0.5 mL) were spread onto 20 mL agar plates and incubated at 37 °C for 18 h, followed by 48 h at 4 °C for RFP maturation. Six 9 mm wells per plate were loaded with 75 µL of 0.1 mg/mL MUmb, AMC, or curcumin (free or micellar formulations), and incubated for 12 h. Fluorescence images were acquired using a UVP BioSpectrum imaging system (UVP, Upland, CA, USA) to detect FRET from the drug to RFP (λ_ex, max_ = 380 nm; λem = 485–655 nm).

#### 2.5.4. Fluorescence Spectroscopy

Fluorescence emission spectra of RFP-expressing *E. coli* suspensions (4 × 10^8^ CFU/mL) were acquired using a Varian Cary Eclipse fluorescence spectrometer (Agilent Technologies, Santa Clara, USA). The excitation wavelength was fixed at λex = 520 nm. This provided quantitative data on RFP expression levels.

FRET efficiency **E** was calculated as [56]

**E** = 1 − F_DA_/F_D_, (1)

where F_DA_ and F_D_ represent the fluorescence intensities of the donor in the presence and absence of the acceptor, respectively. Similarly, F_AD_ and F_A_ represent the fluorescence intensities of the acceptor in the presence and absence of the donor, respectively. On the other hand, E = 1/(1 + (r/R_0_)^6^). The Förster radius, R_0_, is the distance at which 50% of the excitation energy is transferred from the donor to the acceptor.

Therefore, the distance between the fluorophores [56]:

**r** = R_0_ × (1/(F_D_/F_DA_ − 1))^1/6^. (2)

R_0_ was calculated using Equation (3):

(3) $$R_{0}=\sqrt[{6}]{\left(\frac{0.0247\times \kappa^{2}\times \Phi_{D}\times J(\lambda )}{\pi^{5}\times N_{A}\times n^{4}}\right)},$$

where κ^2^ is the orientation coefficient, which may be assumed to be 2/3 in the case of isotropic and randomly oriented donor and acceptor molecules. Φ_D_ is the fluorescence quantum yield of the donor; N_A_ = 6.02 × 10^23^ mol^−1^; n is the refractive index, is equal 1.33 for aqueous medium; and J(λ) represents the integral of the spectral overlap. This was calculated using the following equation:

(4) $$J\left(\lambda \right)=\frac{\int F_{D}(\lambda )\times \varepsilon_{A}(\lambda )\times \lambda^{4}d\lambda }{\int F_{D}(\lambda )d\lambda }$$

where F_D_(λ) is the intensity of donor fluorescence, and ε_A_(λ) is the molar absorption coefficient of the acceptor.

The thermodynamic parameters of drug interactions with amphiphilic polymers were determined using Hill’s linearization in an n-binding site model: lg (θ/(1 − θ)) = n · lg [polymer]—lg *K*_d_, where θ = | (I–I_0_)/(I_∞_–I_0_) |is a fraction of the bound substance. n in the Hill equation is the number of drug molecules per 1 polymer chain.

#### 2.5.5. Interaction of Bioactive Molecules with *E. coli*

The interaction of bioactive molecules (MUmb, curcumin) with *E. coli* was investigated using both 96-well plates and sterile 2 mL Eppendorf tubes. All experiments were performed using a bacterial density of 5 × 10^7^ CFU/mL and a drug concentration of 0.1 mg/mL in a 50:50 (*v*/*v*) mixture of LB broth and PBS (pH 7.4, 0.01 M) at 37 °C. Real-time kinetic fluorescence measurements (λex = 520 nm; λem = 550, 580 nm) were performed using a SpectraMax M5 plate reader (Molecular Devices, CA, USA) with Costar black/clear-bottom 96-well plates. Measurements were taken at the initiation of the interaction and at subsequent time points over 1–2 days to monitor the interaction kinetics.

#### 2.5.6. Assessment of Antibacterial Activity

The antibacterial activity of the bioactive compounds MUmb, AMC, and curcumin against *E. coli* was evaluated using a broth microdilution assay. An overnight *E. coli* culture (grown as described in Section 2.5.1, yielding approximately 4–7 × 10^8^ CFU/mL) was diluted to a working concentration of 5 × 10^6^ CFU/mL. 200 µL of this diluted culture was added to each well of a 96-well plate. A total of 50 µL of serially diluted bioactive compounds were then added to the wells, establishing a range of concentrations for analysis. Bacterial growth was monitored by measuring the optical density (OD_600_) at various time points. To determine the inhibitory concentration (IC), the number of colony-forming units (CFU) was also determined using both OD_600_ measurements and plate counts after appropriate dilutions, ensuring accuracy and consistency. The relationship between CFU and the concentration of each bioactive compound was modeled using a Boltzmann sigmoidal function using (Origin Pro software 2022 V. 9.9.0.225): CFU = (Control CFU)/(1 + exp(([X] – IC_50_)/k)), where [X] represents the concentration of the bioactive compound, IC_50_ represents the half-maximal inhibitory concentration, and k is a constant describing the steepness of the curve. The IC_50_ and IC_90_ values, representing the concentrations at which 50% and 90% inhibition of bacterial growth are observed, respectively, were then determined by fitting this equation.

## 3. Results and Discussion

### 3.1. Article Design

The design of this work encompasses several key aspects:

1. Synthesis of polymeric micelles based on Chit-OA, Chit-LA, Hep-OA, and Hep-LA micelles. Infrared (IR) and nuclear magnetic resonance (NMR) spectroscopic data confirming their structure are presented. The size and morphological characteristics of the micelles, as measured by atomic force microscopy (AFM), are discussed.

2. Drug interaction with micelles: physico-chemical characterization of the interactions between curcumin, MUMb, AMC, and micelles forming polymers, including binding constants and stoichiometry. IR and NMR spectroscopic data confirm the successful integration of the drugs into the micelles, the size and morphology, as assessed by AFM.

3. Visualization of the interaction of micelles with bacteria using AFM. Analysis of morphological changes.

4. FRET analysis of micellar drug interaction with bacteria expressing RFP. Demonstration of RFP (acceptor) fluorescence enhancement and quenching the fluorescence of drugs (donor). Quantification of the interaction.

5. Discussion of the advantages of the developed FRET technique and the prospects of using polymer micelles for the delivery of antibacterial, antioxidant, and anti-inflammatory drugs.

### 3.2. The Synthesis and Spectral Analysis of Chitosan (Chit) and Heparin (Hep) Polymeric Micelles Functionalized with Oleic (OA) and Lipoic (LA) Acid Moieties

**Figure 1:**
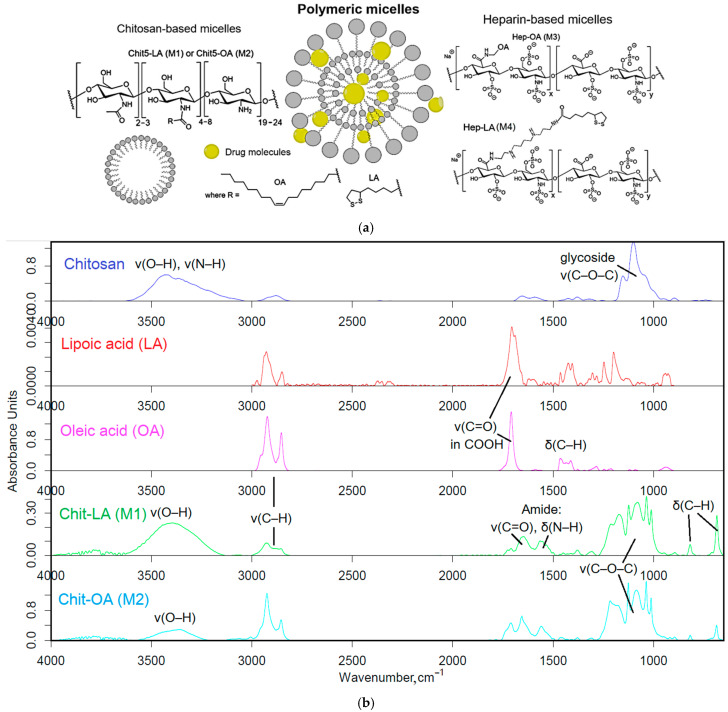

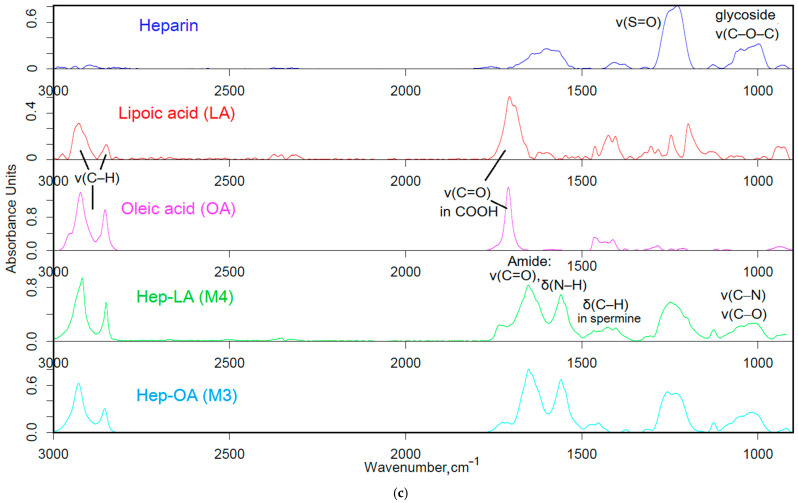
(**a**) Schematics of polymer micelles and structures of modified chitosans and heparins. (**b**) FTIR spectra of chitosan, lipoic acid, oleic acid, and chitosan grafted with oleic acid or lipoic acid residues—M1 and M2. (**c**) FTIR spectra of heparin, lipoic acid, oleic acid, and heparin grafted with oleic acid or lipoic acid residues—M3 and M4.

The polymers (chitosan and heparin) were functionalized with either oleic acid residues for enhanced solubility or lipoic acid residues to impart redox sensitivity to the micelles (Figure 1a). The formation of these micelles occurred in two stages: grafting of polymers and the subsequent formation of micellar systems using an extruder.

Firstly, amphiphilic polymers were synthesized, based on polycationic or polyanionic components and hydrophobic moieties. The synthesis of chitosan and heparin modified by fatty acid is illustrated in Figure S1. The synthesis process is based on activating the carboxyl group of either lipoic or oleic acid, followed by forming a bond with the amine group of chitosan via carbodiimide approach, denoted as M1 and M2. For heparin, the process is reversed, with the carboxyl group from heparin interacting with the amino group from oleyl-amine following a similar mechanism, designated as M3. To create the conjugate of heparin and lipoic acid, Hep-LA (M4), it is necessary to introduce a spermine spacer to the polymer’s amino functionality. This method effectively allows for the attachment of hydrophobic residues to the polymer backbone, resulting in the formation of amphiphilic structures crucial for micelle formation.

The Fourier transform infrared (FTIR) spectroscopy method was employed to validate the successful synthesis of amphiphilic polymers. The analysis of the acquired spectra unveiled critical modifications in the structure of the engineered biopolymers. Figure 1b presents the FTIR spectra of chitosan (Chit5), oleic acid (OA), lipoic acid (LA), and their conjugated products (Chit5-LA and Chit5-OA). A reduction in the signal strength at 1710 cm^−1^, attributed to oscillations in the carboxyl group of acids, is coupled with the emergence of absorption peaks at 1660 and 1560 cm^−1^ corresponding to oscillations in the amide functional group (Amide I and II), indicating the formation of an acid-crosslinking amide bond in chitosan. Additionally, there is a decline in the intensity of a band in the 3600–3200 cm^−1^ range linked to fluctuations in N-H bonds. A modification in the profile of C-O-C glycoside moieties in chitosan reveals the formation of micellar structures featuring hydrophobic and hydrophilic regions, accompanied by a transformation in the conformational state of polymer chains.

In the FTIR spectra of heparin and its derivatives with LA and OA (Figure 1c), prominent bands are observed at 1250 cm^−1^, corresponding to S=O oscillations in sulfate groups SO_3_^−^), and in the frequency range between 1100 and 1000 cm^−1^, representing C-O-C vibrations. Incorporating LA and OA into heparin results in the emergence of Amide I and Amide II bands, indicating the formation of C(=O)-NH amide bonds. The FTIR spectra of conjugate exhibit signals for both initial components and newly introduced functional groups, generally confirming the successful modification of chitosan and heparin through the incorporation of fatty acids.

Table 1 presents the physico-chemical characteristics of amphiphilic conjugates based on chitosan and heparin modified with oleic acid or lipoic acid residues. It demonstrates the effect of the type of polymer and modifying fatty acid on the properties of the conjugates obtained, and their ability to perform micelle formation. It can be seen that the degree of modification of chitosan (Chit5 kDa) by both lipoic (LA) and oleic (OA) acids is quite high (22–25%), which indicates the effectiveness of the inoculation reaction. The average molecular weight of one polymeric structural unit for both conjugates (M1 and M2) is 6.4–6.8 kDa. Positive values of the zeta potential (+12–+15 mV) confirm the cationic character of the obtained chitosan-based micelles. The critical micelle formation concentration (CMC, determined using fluorescent pyrene probe) for Chit5-OA (M2) is lower than for Chit5-LA (M1) (0.33 µg/mL vs. 0.49 µg/mL), which indicates easier micelle formation in the case of the conjugate with oleic acid (Figure S2).

In the case of heparin, the degree of modification by both OA and LA is lower (10–13%) than that of chitosan. This may be due to the peculiarities of the heparin structure and the lower availability of reaction centers. The average molecular weight of the structural unit for Hep-OA (M3) and Hep-LA (M4) conjugates is higher (14 kDa), due to the higher molecular weight of the initial heparin. The negative values of the zeta potential (–7–10 mV) confirm the anionic character of heparin-based micelles. CMC for Hep-OA (M3) is lower than for Hep-LA (M4) (0.42 µg/mL vs. 0.55 µg/mL, Figure S2), which indicates easier micelle formation in the case of heparin conjugate with oleic acid.

In general, the data obtained indicate the successful synthesis of amphiphilic conjugates based on chitosan and heparin, capable of forming micelles under physiological conditions. Differences in CMC for different conjugates may be related to differences in the hydrophobic–hydrophilic balance of molecules, and may affect the efficiency of drug loading and delivery.

### 3.3. Preparation and Characterization of Micellar Formulations of Bioactive Molecules and Adjuvants

The incorporation of bioactive molecules (curcumin, MUmb, and AMC) into polymeric micelles based on chitosan and heparin modified with oleic or lipoic acid residues was carried out using ultrasound treatment followed by extrusion through a membrane with 200 nm pores. This method makes it possible to effectively encapsulate hydrophobic molecules in the core of micelles (Table 2). The type of micelle significantly impacts the degree of drug incorporation for all three substances. Across all micelle types, MUmb exhibits the highest incorporation percentage, consistently exceeding 17% (expressed in mass percentages). This suggests a strong interaction between MUmb and all four micellar structures in the hydrophobic core. Curcumin demonstrates a lower incorporation degree, ranging from 3.5% (for Hep-LA) to 13% (for Chit5-OA). This variability implies that the interaction between curcumin and the micelles is highly dependent on the specific micellar structure. The solubilization of curcumin requires a long fatty acid residue to create a more hydrophobic environment, and it is possible to stabilize the chitosan–curcumin complex due to the formation of hydrogen bonds.

AMC shows moderate incorporation percentages, generally ranging between 13% and 21%, suggesting a relatively consistent interaction with the different micelles, although less pronounced than MUmb, due to the presence of an amino group in AMC and the possibility of repulsion from chitosan cationic chains, and vice versa by stabilization in heparin micelles. OA-containing micelles consistently show high incorporation for curcumin and MUmb, while LA-containing micelles shows low curcumin incorporation. Micelles with a charge opposite to that of the drug’s charged groups are necessary to maintain their stability. This underscores the significance of nature and makeup of polymeric micelles in determining the optimal level of drug loading.

**Figure 2:**
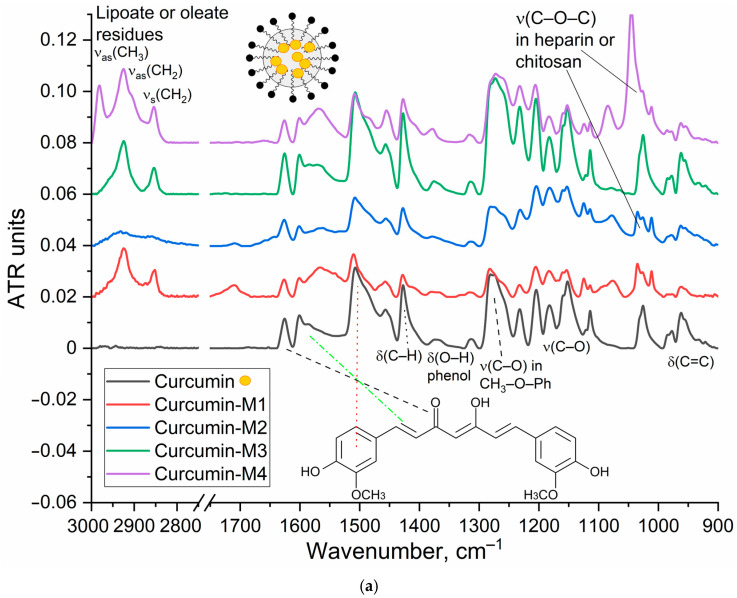

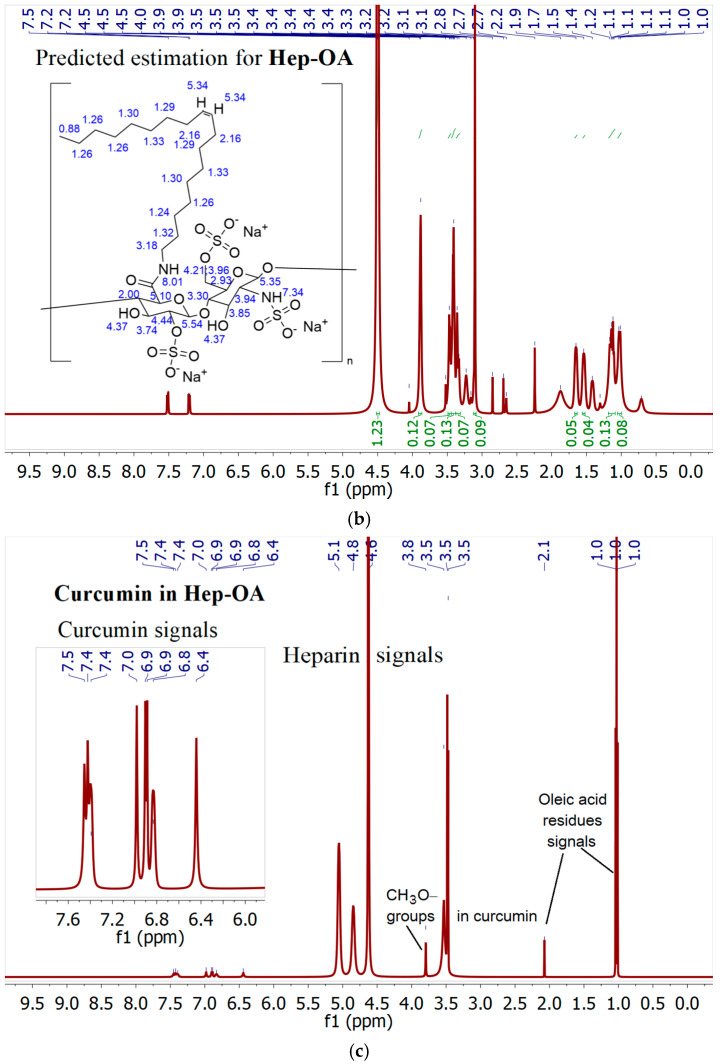

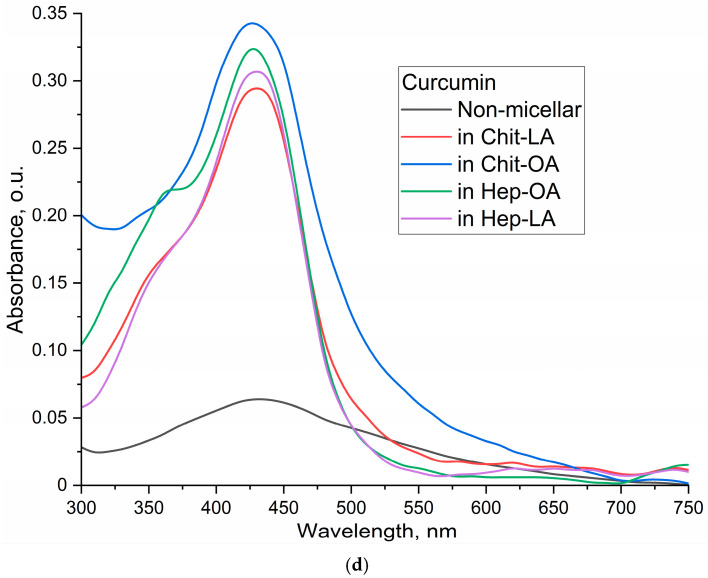
(**a**) FTIR spectra of curcumin in free form and loaded into polymeric micelles M1-M4. PBS (0.01 M, pH 7.4). (**b**,**c**) ^1^H NMR spectra of Hep-OA micelles in D_2_O and Hep-OA micelles loaded with curcumin in D_2_O. (**d**) UV/Vis absorbance spectra of curcumin (5 µg/mL) in non-micellar form (1% DMSO, PBS) and loaded into polymeric micelles. T = 37 °C.

The characterization of the inclusion of curcumin in micelles was carried out by FTIR and NMR spectroscopy. Figure 2a shows the IR spectra of curcumin in free form and loaded into M1–M4 polymer micelles. The inclusion of curcumin in micelles is confirmed by changes in the characteristic absorption bands. There is a broadening and displacement of bands corresponding to v(C=C) oscillations in the composition of aromatic rings (1505 −> 1507–1510 cm^−1^; 1600 −> 1602–1603 cm^−1^), which indicates a change in its environment during encapsulation in the hydrophobic core of micelles. On the other hand, the intensity ratio of peaks I1625/I1505 = I(v(C=O))/I(v(C=C)) changes, which characterizes the change in the microenvironment of carbonyl groups of curcumin in the direction of increasing the degree of hydration, due to interaction with polymer hydrophilic chains. This is further confirmed by a low-frequency shift in the oscillation band of the carbonyl groups of curcumin.

NMR spectroscopy provides data complementary to FTIR spectroscopy. ^1^H NMR spectra of Hep-OA micelles in D_2_O (Figure 2b) show heparin proton signals in the range of 3.0–4.5 ppm, as well as oleic acid proton signals in the range of 0.8–2.5 ppm. The presence of signals in the 5.3 ppm region corresponds to a proton with a double bond in oleic acid (low intensity). ^1^H NMR spectra of Hep-OA micelles loaded with curcumin (Figure 2c), in addition to Hep-OA signals, contain signals of curcumin protons. Characteristic signals of aromatic protons of curcumin are observed in the region of 6.4; 6.8–7.0 and 7.3–7.5 ppm, and the signals of the methoxy groups are about 3.8 ppm. The appearance of these signals clearly confirms the successful encapsulation of curcumin. The low intensity of curcumin signals is associated with the limited solubility of curcumin in micelles (up to 0.5 mg/)mL and the NMR spectroscopy requirements for sample concentration (about 10 mg/)mL. The observed broadening of the signals of curcumin protons may be due to the limitation of their mobility within the micellar structure.

The UV–Vis absorption spectra of curcumin (Figure 2d) reveal a significant impact of micellar encapsulation on curcumin’s solubility and aggregation state. The non-micellar curcumin sample (1% DMSO in PBS) displays a broad, less defined absorption spectrum, indicating the presence of aggregated curcumin particles. This broadness arises from light scattering by larger curcumin aggregates, which are formed due to the poor water solubility of curcumin.

In contrast, the spectra of curcumin loaded into the polymeric micelles exhibit significantly sharper and more defined peaks centered around 420 nm. This indicates a more homogeneous distribution of curcumin molecules, likely due to efficient encapsulation within the micelles, thus preventing aggregation. Using the molar extinction coefficient (ε) of 27,500 M^−1^cm^−1^ for curcumin in ethanol, the calculated concentrations were approximately 0.9 µg/mL for the non-micellar sample and significantly higher for the micellar samples: approximately 4.0 µg/mL in Chit-LA, 4.6 µg/mL in Chit-OA, 4.4 µg/mL in Hep-OA, and 4.1 µg/mL in Hep-LA (at a 0.1mg/mL concentration). These concentrations, combined with the spectral data, strongly support the conclusion that the micelles substantially enhance curcumin’s solubility and prevent aggregation in aqueous solution. The differences between micellar formulations reflect their varying solubilizing capacities, with Chit-OA and Hep-OA demonstrating superior performance. The observed spectral differences—a broad, less-defined spectrum for aggregated non-micellar curcumin versus a sharp, well-defined spectrum for solubilized micellar curcumin—provide strong evidence of the effectiveness of the micelles in enhancing curcumin’s solubility.

Thus, the effective encapsulation of aromatic medicinal biomolecules (using curcumin as an example) into polymer micelles is shown.

### 3.4. Visualization of the Polymeric Micelles and Its Interaction with E. coli Using AFM

**Figure 3:**
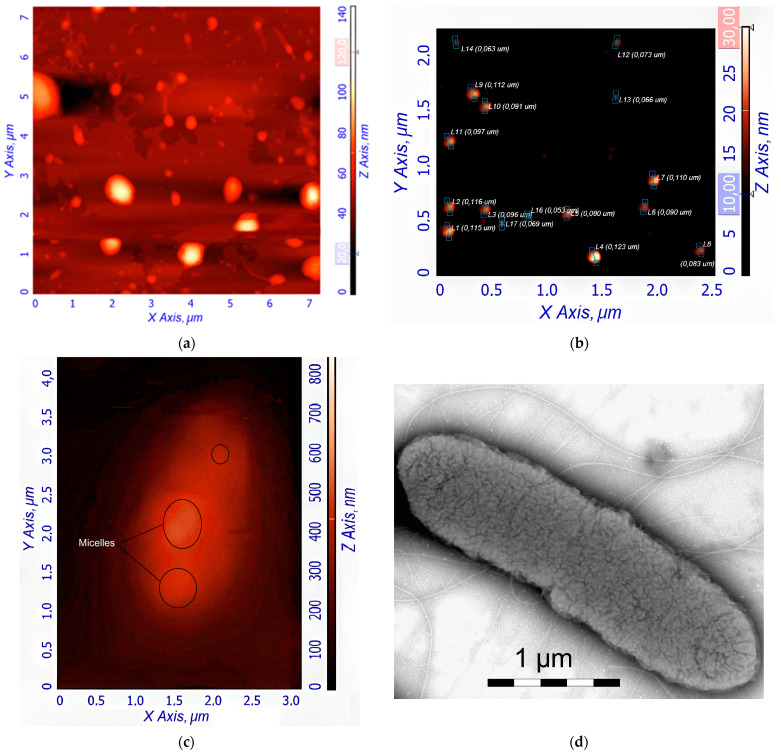

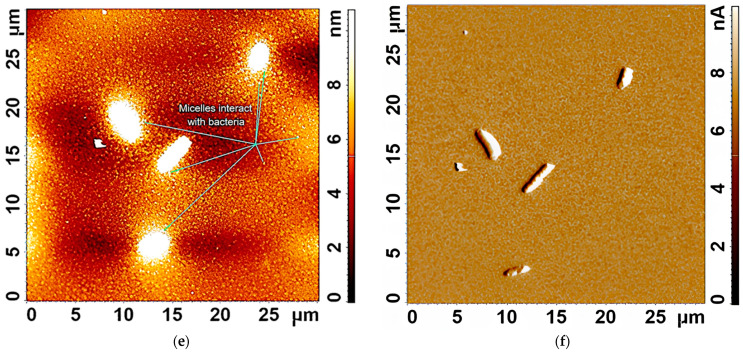
AFM images of (**a**) Hep-OA empty micelles and (**b**) Hep-OA micelles loaded with curcumin. (**c**) AFM image of single *E. coli* cell pre-incubated with Hep-OA micelles loaded with curcumin. (**d**) TEM image of single *E. coli* cell. (**e**,**f**) AFM images of *E. coli* cells pre-incubated with Chit-LA empty micelles: (**e**) Focusing on the substrate reveals spherical micellar particles. (**f**) Magnitude signal.

Figure 3a,b shows atomic force microscopy (AFM) images of empty Hep-OA micelles (a) and Hep-OA micelles loaded with curcumin (b). AFM makes it possible to visualize the morphology of micelles, determine their size, and evaluate sample homogeneity. The images of empty Hep-OA micelles (Figure 3a) show formations that are far from spherical in shape, with a wide size distribution from 100 to 800 nm. This may indicate the aggregation of micelles and the absence of a well-defined structure in the absence of a loaded substance. The inclusion of curcumin leads to a significant change in the morphology of micelles (Figure 3b). The characterization of the curcumin-loaded Hep-OA micelles revealed a uniform population of spherical particles with a size distribution ranging from 65 to 120 nm. Importantly, no significant aggregation was detected, indicating a high degree of micellar stability and homogeneity. This uniform size and lack of aggregation are crucial factors for ensuring consistent drug delivery and predictable biological interactions.

This change in morphology and decrease in particle size when loaded with curcumin can be explained by the factor that curcumin, being a hydrophobic molecule, is localized in the core of micelles, stabilizing their structure and contributing to the formation of more compact spherical formations. Thus, curcumin plays an important role not only as an encapsulated substance, but also as a structure-forming agent that affects the morphology and size of Hep-OA micelles. Reducing the size and increasing the homogeneity of micelles when loaded with curcumin is important for increasing the efficiency of drug delivery, as it improves their pharmacokinetic properties and bioavailability.

To visualize the micelles interactions with *E. coli*, atomic force microscopy (AFM) and transmission electron microscopy were employed. Figure 3c–f provides visual evidence of the interaction of *E. coli* bacterial cells with the polymeric micelles under study, obtained using these methods. Figure 3c shows an AFM magnified image of a single *E. coli* cell after pre-incubation with curcumin-loaded Hep-OA micelles. The image shows the adsorption of micelles on the surface of a bacterial cell. This indicates the interaction of micelles with the bacterial cell wall, which is important for the subsequent penetration of curcumin into the cell. Figure 3d–f demonstrates the interaction of several *E. coli* cells in a large field with empty Chit-LA micelles. In Figure 3d, spherical micelle particles are visible when focused on the mica. This confirms that Chit-LA micelles retain their morphology and are not destroyed by interaction with bacteria. Figure 3e (height signal) and 3f (magnitude signal) provide additional information about the topography and mechanical properties of the sample. Micelles are partially visible on the surface of bacterial cells. Based on the analysis of the available data, chitosan presented in the form of Chit-LA micelles exhibits remarkable properties in its interaction with *E. coli* cells. Upon contact with bacteria, chitosan maintains its morphological integrity, indicating its durability and potential as a delivery agent for pharmaceuticals. While heparin also exhibits a strong interaction with cells, the adhesion of chitosan is more stable due to electrostatic forces. Consequently, it can be inferred that chitosan may prove to be a more advantageous option in this context, albeit heparin possesses its own unique advantages.

In general, the AFM data indicate the interaction of the studied polymer micelles with *E. coli* bacterial cells. The visualization of this interaction is an important step for understanding the mechanisms of drug delivery using micelles and optimizing their composition to increase the effectiveness of therapy.

### 3.5. Fluorescence Studies of Drug Encapsulation in Polymeric Micelles

**Figure 4:**
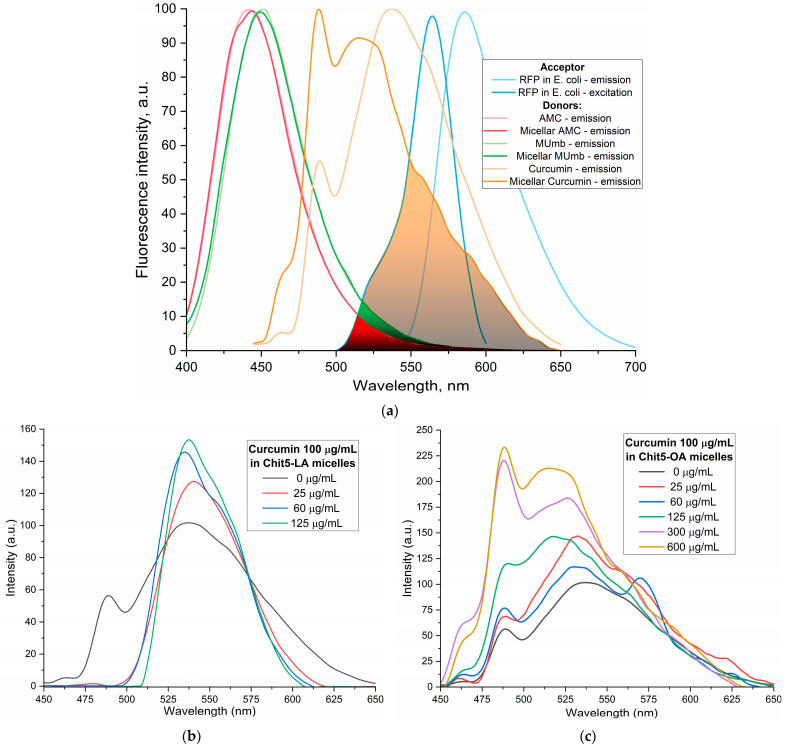

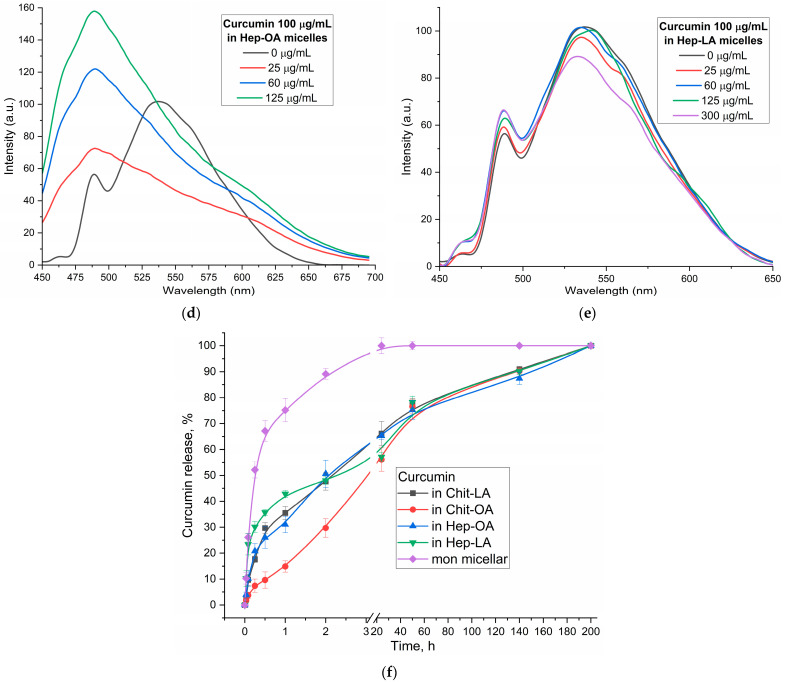
(**a**) FRET pairs. Normalized fluorescent emission spectra of FRET donors: curcumin, MUmb, AMC in free form and in micellar Chit5-OA formulations. Normalized fluorescent spectra of FRET acceptor RFP in *E. coli* cells. Fluorescent spectra of curcumin in free form and in micellar formulations: (**b**) Chit5-LA, (**c**) Chit5-OA, (**d**) Hep-OA, (**e**) Hep-LA. (**f**) Curcumin release profiles. The dialysis membrane, with a cut-off size of 12–14 kDa, was immersed in an external solution, with a ratio of 1:5 by volume. The presence of curcumin was determined by measuring its absorption at 550 nm. The initial concentration of curcumin was set at 0.1 mg/mL, while the initial concentration of the amphiphilic polymer was 1 mg/mL. The results are presented as the mean ± standard deviation of three experiments. PBS (0.01 M, pH 7.4). T = 37 °C.

Curcumin, MUmb, and AMC were selected as model bioactive molecules and suitable fluorescence donors for RFP-based FRET studies. Figure 4a shows the fluorescence emission spectra of curcumin, MUmb, and AMC, both in free form and encapsulated within Chit5-OA micelles, alongside the emission spectrum of the RFP acceptor in *E. coli* cells. The data reveal that encapsulation into the micelles minimally alters the spectral profiles of MUmb and AMC, primarily resulting in a reduction in fluorescence intensity. Table 3 presents data on spectral overlap integrals (J) and Förster radii (R_0_) for RFP-drug FRET pairs. While the encapsulation of curcumin and MUmb within Chit-OA micelles leads to a reduction in the spectral overlap integral (J), a concomitant increase in the Förster radius (R_0_) is observed. This is due to the fact that the reduction in spectral overlap is counterbalanced by the enhancement in the fluorescence quantum yield of the drug donor. This effect is particularly pronounced in the case of curcumin, which significantly influences the efficiency of energy transfer. This increase in R_0_, coupled with a likely enhancement in the fluorescence quantum yield of the encapsulated donor molecules (due to improved environmental shielding within the micelle), could result in an overall increase in FRET efficiency. Consequently, despite the smaller J values, the enhanced R_0_ and increased donor fluorescence could lead to a greater absolute fluorescence intensity from the RFP acceptor. The unchanging FRET parameters for AMC are likely within experimental error. Further quantitative assessment of FRET efficiency, such as fluorescence lifetime imaging microscopy (FLIM) or sensitized emission measurements, is warranted to definitively elucidate the net effect of micellar encapsulation on energy transfer.

Figure 4b–e illustrates the fluorescence spectra of curcumin in its free form and within various micellar formulations. In Chit5-LA micelles (Figure 4b), an increased fluorescence intensity at 535–540 nm is observed with minimal spectral profile alteration, indicating enhanced solubility and a minor change in the curcumin’s microenvironment. In Chit5-OA micelles (Figure 4c), a dramatic spectral shift occurs as a new peak emerges at 485 nm (compared to the 535 nm peak in free curcumin), representing a significant change in the spectral profile. This profound alteration highlights curcumin’s high sensitivity to the hydrophobic microenvironment of the micelle core, making a promising test system for numerous analytical approaches. Curcumin inclusion into OA-containing micelles (Chit5-OA and Hep-OA, Figure 4c,d) demonstrates a sharp change in the curcumin microenvironment and its transition to the hydrophobic core of micelles. This is consistent with previously published data on the dependence of the spectral characteristics of curcumin on the solvent [57]. An increase in fluorescence intensity also indicates an increase in the curcumin solubility (from 0.009 to 0.15 mg/mL) in the presence of micelles. The dissociation constants of curcumin complexes with micelles Chit5-OA and Hep-OA are estimated as 10 and 3 µM, correspondingly.

In the case of LA-containing micelles, curcumin’s fluorescence spectra show less pronounced changes (Figure 4b,e). While a slight fluorescence intensity increase is observed, the emission maximum shift is less significant than in oleic acid-based micelles. This curcumin solubilization up to 0.1 mg/mL is achieved within these micelles, but with less alteration of the microenvironment. The shorter length and terminal thiol group of lipoic acid, imparting less hydrophobicity compared to oleic acid, likely leads to curcumin occupying not only the hydrophobic core but also more hydrophilic regions of the lipoic acid micelles. This is supported by estimated dissociation constants of ~20 and 8 µM for curcumin complexes with Chit5-LA and Hep-LA micelles, correspondingly.

Figure 4f presents the curcumin release profiles, showing a significant difference in release kinetics between the non-micellar curcumin and the various micellar formulations. The non-micellar curcumin exhibits a very rapid release, with a half-release time of only 0.23 ± 0.05 h. This indicates that in the absence of a delivery vehicle, curcumin is readily released into the surrounding environment. In contrast, all the micellar formulations demonstrate a substantially slower release rate. The half-release times for Chit-LA, Chit-OA, Hep-OA, and Hep-LA are 2.2 ± 0.1, 3.0 ± 0.2, 2.0 ± 0.1, and 2.1 ± 0.3 h, respectively. This sustained release is a desirable characteristic for drug delivery, as it can lead to increased efficacy and reduced dosing frequency. The observed differences between the micellar formulations themselves are relatively subtle, suggesting that the choice of polymer and modification (oleic acid vs. lipoic acid) has a relatively small effect on the overall release rate of curcumin in this specific context. The data support the conclusion that encapsulation within these polymeric micelles provides a controlled release mechanism for curcumin, extending its availability and potentially improving its therapeutic effect.

The discussion initially focused on curcumin due to its inherent interest as a bioactive molecule and its complex behavior within the micellar systems. However, to validate the observed effects and investigate the generality of FRET efficiency modulation by micelles, the study was expanded to include Mumb and AMC. These compounds were chosen as they offer superior fluorescence quantum yields compared to curcumin, representing more established fluorophores for FRET applications. Furthermore, AMC, possessing additional anti-inflammatory properties, presents a promising candidate for drug delivery. The inclusion of Mumb and AMC allows for a comparative analysis of FRET efficiency across different fluorophores, ensuring robustness of the findings and expanding the potential therapeutic applications of this micellar drug delivery system.

**Figure 5:**
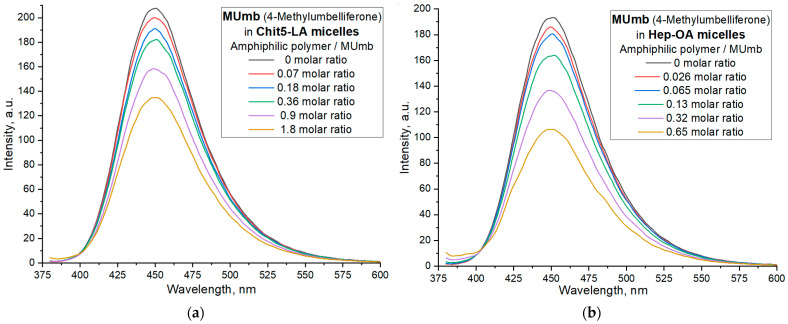

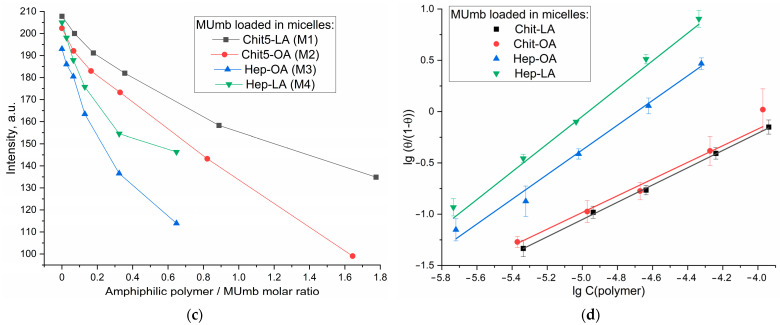
Fluorescent spectra of MUmb in free form and in micellar formulations: (**a**) Chit5-LA, (**b**) Hep-OA. (**c**) Curves of the dependence of the maximum intensity of MUmb fluorescence on the concentration of the added polymer. (**d**) Linearization of (**c**) curves in Hill coordinates. PBS (0.01 M, pH 7.4). T = 37 °C. Hill’s linearization in n-binding site model: lg (θ/(1 − θ)) = n · lg [polymer]—lg *K*_d_, where θ = | (I–I_0_)/(I_∞_–I_0_) |is a fraction of the bound substance. n in Hill equation—number of drug molecules per 1 polymer chain.

Figure 5 illustrates the interaction of 4-methylumbelliferone (Mumb) with the polymeric micelles studied. The fluorescence spectra of Mumb in free form and in the Chit5-LA and Hep-OA micellar systems (Figure 5a,b) demonstrate changes in fluorescence intensity upon addition of polymers. When the fluorophore is incorporated into the micelles, fluorescence quenching is observed; in the case of OA-containing polymers, a stronger quenching is observed than in the case of LA. The sigmoidal curves character of the dependence of the intensity of Mumb fluorescence on the polymer concentration (Figure 5c) is typical for binding processes. The linearization of these curves in Hill coordinates (Figure 5d, Table 4) allowed us to determine the physico-chemical parameters of the interaction of Mumb with micelles, including the apparent dissociation constant (*K*_d_), the binding constant (*K*_A_), and the Hill coefficient (n). *K*_A_ is the concentration of the ligand at which half saturation is achieved, and the Hill coefficient (n) characterizes the cooperativeness of binding.

The obtained values of –lg *K*_d_ show that MUmb interacts more efficiently with heparin-based micelles (Hep-OA and Hep-LA) than with chitosan-micelles (Chit-LA and Chit-OA) by about an order of magnitude in terms of constants. The Hill coefficient (n) for all systems is close to 1, which implies the absence of cooperativity in the binding of MUmb to micelles and the stoichiometry of 1 amphiphilic polymer to 1 MUmb molecule. A slight excess of the n value for Hep-OA and Hep-LA (1.20 and 1.35, respectively) may indicate some positive cooperativeness. High R-square values (close to 1) for all linearizations confirm the adequacy of the Hill model for describing the interaction of MUmb with the micelles.

The fluorescence data indicate the effective incorporation of MUmb into the polymer micelles, but unlike curcumin, we observe fluorescence quenching (due to the incorporation of MUmb in the micellar core) as opposed to ignition (due to the solubilization of curcumin). This opens up prospects for using these systems as carriers for the delivery of MUmb, curcumin, and other hydrophobic drugs.

### 3.6. FRET Technique for Monitoring the Interaction of Fluorophores with Bacterial Cells Express RFP

#### 3.6.1. Visualization of the Interaction of Cells with Drugs

**Figure 6:**
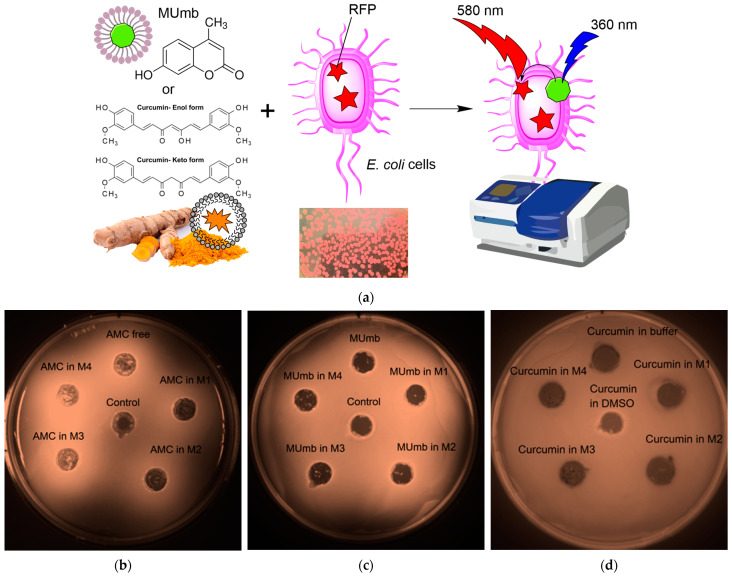
(**a**) Experimental scheme for studying the interaction of fluorophores with bacterial cells expressing RFP using the FRET technique. (**b**–**d**) Petri dish experiment using bacteria expressing red fluorescent protein (RFP) as a visual platform for assessing the permeability of 0.1 mg/mL formulations of AMC, MUmb, and curcumin, both free and encapsulated in micelles. Fluorescence detection on a bioluminescent visualizer. For curcumin, a DMSO sample with a concentration of 1 mg/mL was also used as a positive control. The negative control was PBS (0.01 M, pH 7.4). T = 37 °C.

The proposed FRET based technique to investigate the interaction between fluorophores and bacterial cells expressing red fluorescent protein (RFP) represents a powerful approach for evaluating the permeability of biologically active molecules into the cells. The experimental design depicted in Figure 6a involves incubating RFP bacterial cells with fluorophores or the micelles form, followed by the acquisition of fluorescence spectra compared to cells without incubation with fluorophore. The efficacy of FRET is determined by the activation of RFP fluorescence, which is influenced by the permeability of the bioactive molecules within the cell membrane and their level of interaction with RFP.

The Petri dish experiment, employing bacteria expressing RFP, provided a visual (fluorescence detection) platform to assess the permeability of AMC, MUmb, and curcumin—both free and encapsulated within micelles (Figure 6b–d; Table 5). The RFP-expressing bacteria served as a dynamic backdrop, highlighting the diffusion of fluorescent compounds. Table 5 presents RFP fluorescence intensity measurements from a Petri dish assay using *E. coli* expressing RFP to assess the cellular permeability of drugs. The data show variable effects of the different formulations on RFP fluorescence intensity (as quantitative values of FRET).

Curcumin, despite its known bioactivity, exhibited low fluorescence signal, due to its inherently low quantum yield. The weakly expressed fluorescent halo around the curcumin-containing wells (Figure 6d) suggests poor membrane permeability in its free form, highlighting a necessitates a delivery system for its therapeutic application.

In contrast, both MUmb (Figure 6c) and AMC (Figure 6d) demonstrated a clear fluorescent halo, indicating successful penetration into the surrounding bacterial medium. This suggests that these compounds, at least in their free forms, possess greater intrinsic membrane permeability than curcumin. Crucially, the experiment also revealed a striking dependence on the micelle type: MUmb achieved maximal penetration when delivered via chitosan micelles (the diameter of the halo is about 30 mm vs. 25 mm for other formulations), while AMC showed superior performance with heparin micelles (the diameter of the halo is about 25 mm vs. 20–21 mm for other formulations).

This differential response to micelle type strongly implies that the choice of carrier system is paramount for optimizing drug delivery. The specific chemical interactions between the micelle material and the bacterial membrane appear to play a crucial role, and these results highlight the potential for targeted drug delivery approaches by selecting micelles with enhanced compatibility with specific cell types. This simple Petri dish assay thus offers a powerful visual and quantitative method for assessing the efficacy of different drug delivery strategies.

#### 3.6.2. Spectral Analysis of the Interaction of Cells with Drugs

**Figure 7:**
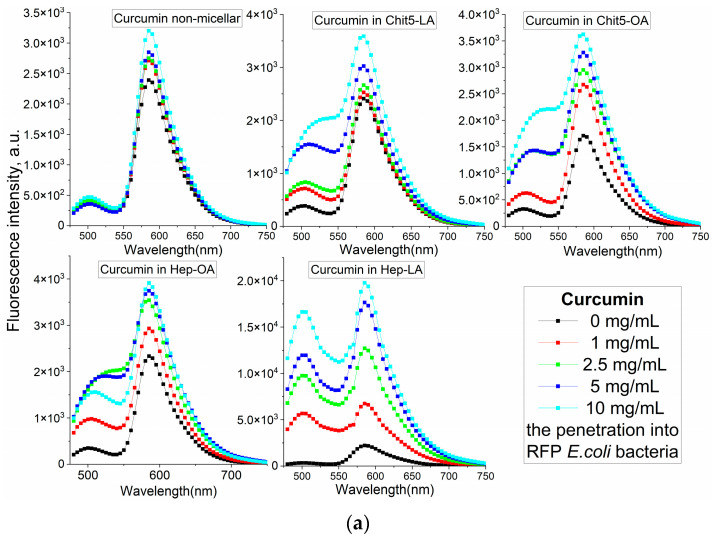

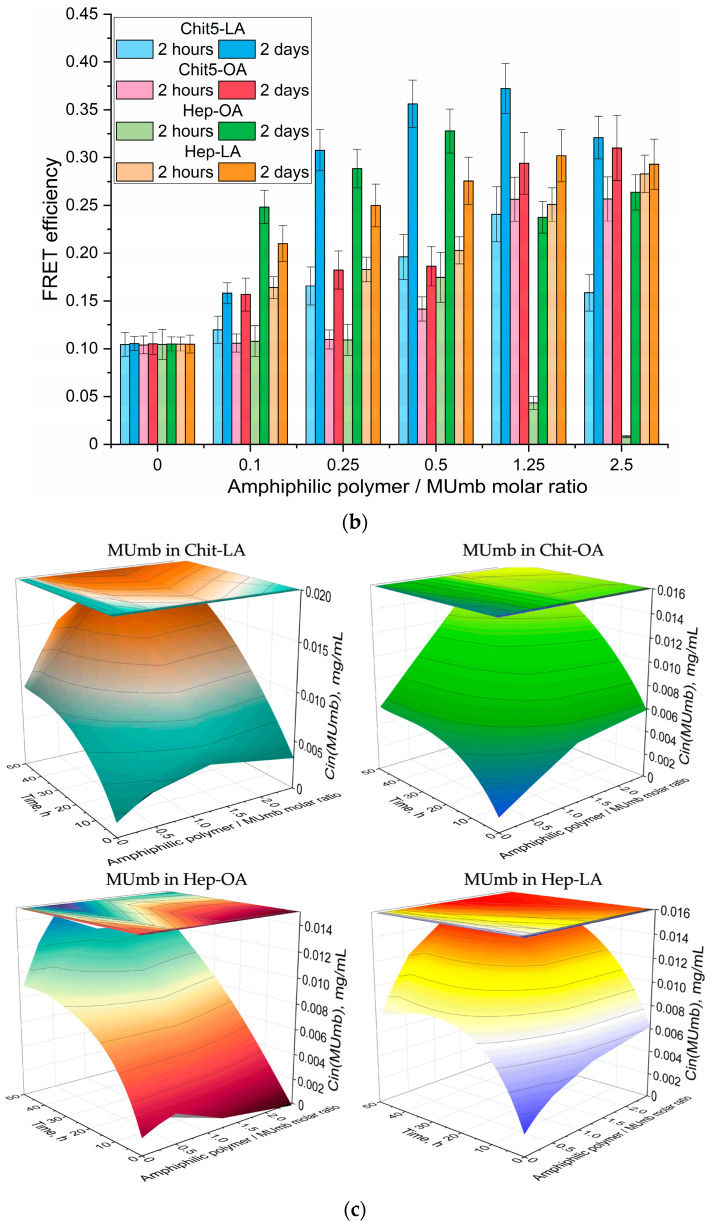
(**a**) Fluorescent spectra of a suspension of *E. coli* bacteria (5 × 10^7^ CFU/mL) with added curcumin in various concentrations and compositions. (**b**) The effectiveness of FRET between MUmb (0.1 mg/mL) and RFP as a function of the time of micelle type and concentration of the amphiphilic polymer. (**c**) Dependence of the concentration of MUmb (0.1 mg/mL) inside bacterial cells on the incubation time of the drug with cells and the composition of the formulation. FRET-derived estimation of intracellular MUmb was validated by independent measurements obtained after cell lysis and subsequent fluorometric quantification of MUmb. PBS (0.01 M, pH 7.4). T = 37 °C.

The analysis of the fluorescent spectra in Figure 7 provides more detailed information on the interaction between drugs and cells in a more formal context. In this segment, we could determine how various conditions, and the composition of micelles impact the fluorescent properties of the drugs under investigation.

The increase in the fluorescence intensity of RFP fluorescence by approximately 40% upon the addition of free curcumin suggests its penetration into the cells and interaction with RFP (Figure 7a). However, the employment of micellar formulations markedly enhances this effect. There is a significant difference in the efficacy of FRET for various types of micelles, namely Chit5-LA, Chit5-OA, Hep-OA, and Hep-LA. This variation indicates the influence of both the polymer type and the modifying fatty acid on the permeability. Micelles based on chitosan with oleic acid, Chit5-OA, exhibit a remarkable enhancement of RFP (115%). This suggests a superior ability of these micelles to penetrate bacterial cells owing to their effective solubilization of curcumin. Curcumin in Hep-LA micelles exhibit a remarkable degree of RFP enhancement, approximately 700%. This can be due to the electrostatic interactions between heparin and the positively charged RFP. In addition, the heparin facilitates the process of drug penetration into the cell. Chit5-LA and Hep-OA demonstrate an intermediate level of efficiency, with 50% and 75% RFP activation, respectively.

Micellar curcumin’s intracellular fate involves: (1) membrane binding; (2) endocytosis (type depends on micelle properties); (3) curcumin release (mechanisms include lysosomal acidification or enzymatic degradation); and (4) intracellular distribution (including potential mitochondrial targeting due to curcumin’s hydrophobicity).

Fluorescence resonance energy transfer (FRET) allowed us to investigate the penetration of drugs into bacterial cells and the kinetics of fluorescence penetration, contingent upon the presence and composition of micelles. Figure 7b presents the data on the FRET dynamics of MUmb over a period of two hours and 48 h, revealing an increase in FRET efficacy over time, and indicating a gradual accumulation of fluorescent molecules within cells.

After two hours of incubation, micelle penetration remained relatively slow, reaching a maximum of approximately 15%. This limited penetration may be attributed to the prolonged interaction of MUmb-loaded micelles with cellular structures and their slower diffusion rates compared to the free MUmb. Furthermore, the encapsulation of MUmb within the micellar matrix may limit its immediate bioavailability. A control experiment using the free MUmb clarified the contribution of micellar encapsulation to the observed slower penetration rate.

Following 48 h of incubation, fluorescence resonance energy transfer (FRET) is predominantly driven by fluorophores that have successfully internalized in the cells. For most micelle types, the FRET efficiency reaches a plateau at approximately 0.3, significantly surpassing the performance of non-micellar MUmb, which exhibits an efficiency of 0.1 and practically fails to penetrate bacterial cells and the drug interacts poorly with RFP without micelles. This observation underscores the efficacy of micellar drug delivery in enhancing the intracellular distribution of therapeutic agents.

In summary, we are indeed observing FRET, particularly for curcumin (as shown in Figure 7a). In micelles, the intensity of FRET is enhanced, possibly due to the concentration enhancement effect. In the solution, we could not detect FRET, as we noted in our earlier article [58]; it is only observable in cells and micellar systems. Our results highlight that the type of polymer used (either chitosan or heparin) and the modifying fatty acid (such as oleic or lipoic) significantly influence the permeability of these micellar formations into bacterial cells. Micelles containing lipoic acid residues result in an average higher FRET efficiency compared to OA-containing micelles. This is due to the smaller size of the former and the greater hydrophilicity, which is important for the interaction with RFP. Heparin-based micelles exhibit the best permeability, which makes them promising for drug delivery into bacterial cells. The high efficiency of Chit5-OA also deserves attention. Therefore, both types of these micelles can be used to deliver other hydrophobic drugs, including anticancer drugs, antibiotics and antiviral agents. Figure 7bc illustrates the intracellular MUmb concentration in *E. coli* cells as a function of exposition time (2 h vs. 2 days), and as a function of the molar ratio of amphiphilic polymer to drug (MUmb). This figure demonstrates the time-dependent accumulation of drug and the influence of the polymer-mediated delivery system on its intracellular concentration. We evaluated intracellular MUmb concentrations in E. coli using a FRET-based approach by using the correlation of the FRET efficiency with drug intracellular concentrations obtained in the independent experiment. This FRET-derived estimation of intracellular curcumin was validated by independent measurements obtained after cell (without RFP) lysis and subsequent fluorometric quantification of MUmb (or curcumin).

At the 2 h incubation time, the intracellular MUmb concentration is relatively low and shows only a marginal increase with an increasing polymer:MUmb molar ratio, particularly for M1 and M2 formulations. This suggests a limited uptake of MUmb within this short timeframe, regardless of the formulation used. However, a significant difference is observed after a 2-day incubation. An increase in intracellular MUmb concentration is observed, demonstrating time-dependent cellular uptake. The effect is most pronounced for M3 (Hep-OA) formulation. At higher polymer:MUmb ratios, M3 shows substantially higher intracellular curcumin levels compared to other formulations after 2 days. This indicates that M3 facilitates significantly MUmb delivery and uptake into bacterial cells over a longer period. M2 (Chit5-OA) also shows enhanced MUmb uptake after 2 days, although less pronounced than observed for M3. The Hep-OA (M3) formulation appears particularly effective in mediating MUmb uptake after prolonged incubation, suggesting a potential advantage for this formulation in antimicrobial applications.

The FRET-based approach suggested here provided robust quantification, minimizing potential errors inherent in either technique alone. Crucially, our FRET-based measurements are in agreement with our prior work on the permeability of some other drug such as doxorubicin, moxifloxacin, eugenol into the cells [59,60]. This measurement of the drug concentration in the supernatant after cell lysis (with Triton X100), provided only an endpoint result. Even after 14 h of drug incubation with *B. subtilis*, a significant portion of drug remained associated with the cell wall (40 µM) and only a small amount was taken up by the cells (0.5 µM). Notably, the lower sensitivity of the lysis method (compared to our FRET method), which yields only endpoint measurements, hinders the ability to capture the kinetics of the drug interaction with cells. In contrast, FRET methodology allows for real-time observation of MUmb interactions, providing a much more comprehensive understanding of the uptake and cellular processing of the substance compared to the previous lysis-based method.

The use of polymeric micelles as drug delivery vehicles offers several key advantages. The pharmacokinetic (PK) properties of amphiphilic polymer micelles (M1 and M2) loaded by model drug-fluorophore doxorubicin (Dox) have been previously characterized in Wistar rats (Table S1, reproduced from [41]). As shown, micellar formulations (particularly M2) exhibited prolonged half-elimination times compared to free Dox and showed increased area under the curve (AUC) values, indicating enhanced micellar drug exposure. These PK results suggest improved drug delivery characteristics of these micellar systems. Encapsulation within micelles can significantly improve the physicochemical properties of poorly soluble drugs, such as curcumin, enhancing their bioavailability and therapeutic efficacy. Micelles protect the encapsulated drug from degradation, increasing its stability in physiological environments. Furthermore, the targeted delivery potential of micelles, achieved through surface modifications or ligand conjugation, allows for increased accumulation of the drug at the desired site of action, while minimizing off-target effects and systemic toxicity.

### 3.7. Antibacterial Activity of Curcumin, MUmb, and AMC in Free Form and in Micelles as Independent Substances and Adjuvants for Antibiotics

The employment of polymeric micelles as nano-carriers for the transportation of curcumin, MUMb, and AMC presents promising avenues in the battle against infections. These compounds are known for their anti-inflammatory and antibacterial properties; however, their limited solubility in aqueous solutions and rapid metabolic degradation pose constraints on their clinical application. The micellar delivery system addresses significant challenges by enhancing solubility, increasing bioavailability, and facilitating the controlled release of active components. Regarding the inhibition of efflux proteins, we are developing a formulation that simultaneously incorporates an antibiotic agent and an adjuvant to suppress the activity of efflux pumps, which are one of the primary mechanisms of multidrug resistance. This approach aims to improve the efficacy of antibiotic treatments by overcoming one of the key barriers to their effectiveness.

So, the antimicrobial activity of curcumin, MUmb, and AMC were assessed both in their free form and incorporated into various micellar systems, including Chit5-LA, Chit5-OA, Hep-OA, and Hep-LA. Additionally, their combined effects with moxifloxacin were explored. The findings are presented in Table 6, which illustrates the percentage of viable cells following treatment with the samples at a concentration of 0.1 mg/mL.

A comprehensive analysis of the data presented in Table 5 reveals that all three substances exhibit antimicrobial activity with comparable efficacy values. Notably, the encapsulation within micelles significantly influences their potency. For instance, curcumin demonstrates the highest activity in Hep-OA (M3), owing to the enhanced solubility and cellular penetration of these particles. Conversely, MUMb exhibits the most pronounced effect in Chit5-OA (M2), which is attributed to its solubilization through OA residues. These findings underscore the critical importance of selecting the appropriate micelle type for the successful delivery and controlled release of each compound.

**Figure 8:**
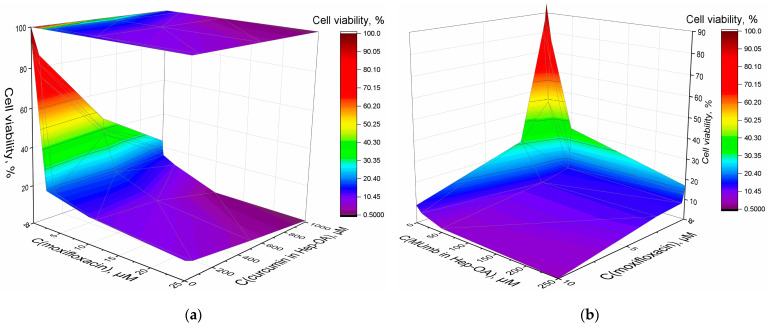
The effect of (**a**) curcumin and (**b**) MUmb on the synergistic enhancement of the antibacterial activity of moxifloxacin against *E. coli* cells. The initial values were 2 × 10^7^ CFU/mL. T = 37 °C.

Furthermore, we explored the synergistic impact of the combined application of curcumin, MUmb, and moxifloxacin, as depicted in 3D diagrams (Figure 8a,b), that show the dependence of bacterial cell viability on the concentrations of moxifloxacin and each of the adjuvants. The most important feature is a significant increase in the antibacterial effect of moxifloxacin at low concentrations (up to 5 µM—pharmacological relevant values) in the presence of curcumin and MUmb. This enhancement reaches an impressive 40–50%, which indicates the high effectiveness of combination therapy. At low concentrations of moxifloxacin, its intrinsic antibacterial activity is limited. However, the addition of curcumin or MUmb significantly increases the effectiveness of moxifloxacin, leading to a significant decrease in bacterial cell viability. This means that curcumin and Mumb do not just add their effects to moxifloxacin. They act together and render the antibacterial effect more efficient.

This enhanced antibacterial efficacy may be attributed to several mechanisms:

1. Inhibition of efflux pumps. Both curcumin [11,61], umbelliferones [62] and coumarins [63] have the ability to inhibit bacterial efflux pumps. These pumps are responsible for expelling antibiotics from bacterial cells, thereby reducing their effectiveness. By blocking these pumps, moxifloxacin can exert its action more effectively.

2. Increased membrane permeability. The use of micellar formulations, particularly those incorporating oleic acid, presumably, enhances the permeability of bacterial membranes. Micellar effect was shown by us earlier in the work [42].

3. Modification of microbial environment. The presence of micelles alters the microenvironment within bacterial cells, potentially amplifying the effects of moxifloxacin.

The synergistic interaction between curcumin and moxifloxacin represents a valuable finding. By enhancing the efficacy of existing antibiotics through combined therapy, we can mitigate the risk of antibiotic resistance and broaden the range of available treatments for infections resistant to conventional approaches. This is particularly significant in the context of the worldwide problem of antibiotic resistance. These data contribute to advancements in personalized medicine, enabling the selection of optimal drug combinations and delivery systems that maximize therapeutic outcomes while minimizing adverse reactions.

## 4. Conclusions

The FRET-based platform allows for a quantitative evaluation of intracellular delivery of bioactive compounds (AMC, MUmb, and curcumin) into bacterial cells using polymeric micelles, leveraging the inherent properties of RFP expressed in *E. coli*. Typically, the assessment of intracellular delivery could include a fluorophore, loaded in delivery vehicle, with subsequent analysis using confocal microscopy, or other relevant methods. However, fluorescence can be quenched by a non-specific interaction with intracellular proteins, or the fluorophore can absorb on the cell surface instead of intracellular delivery, which is very difficult to distinguish by spectroscopic methods. The use of a test compound (or drug) as FRET donor, and RFP as acceptor, allows for direct visualization via FRET effect only when the interaction takes place inside the cell, making it a much more specific approach.

The FRET effect was robust throughout the three different donor compounds and different compositions of the polymeric micelle, enabling the possibility to select the most efficient vehicle. This assay visually demonstrated superior cell membrane permeability for polymeric formulations of AMC and MUmb, and poorly permeability curcumin. Specifically, a 18–30% increase in fluorescence AMC intensity was observed for curcumin delivered via Hep-OA and Hep-LA micelles compared to free drug, indicating the enhanced FRET efficiency. The optimal micelle type varied depending on the drug: MUmb showed maximal penetration with chitosan micelles, while AMC exhibited the highest penetration with heparin micelles.

Micellar formulations exhibited enhanced antibacterial activity compared to free drugs, with Hep-OA micelles demonstrating the most significant reduction in *E. coli* viability. Synergistic effects were observed when combining curcumin and MUmb with moxifloxacin, resulting in a remarkable 40–50% increase in efficacy, particularly at low (≤5 µM) moxifloxacin concentrations. This synergistic effect suggests that curcumin and MUmb not only contribute additively but also potentiate moxifloxacin action, potentially allowing for a 2–3-fold reduction in moxifloxacin concentration to achieve the same antibacterial effect.

We have combined the sensitivity and precision of FRET with the simplicity of a Petri dish assay for real-time visualization and antibacterial activity assessments. This study offers a robust platform for optimizing micelle-based drug delivery systems and evaluating their antimicrobial effectiveness. The key advancement is the direct visualization and quantitative measurement of intracellular drug delivery, providing insights into micellar interactions with bacteria at the nanoscale, impossible with traditional methods. This method promises to develop targeted therapies against bacterial infections.

Future directions include expanding this platform to investigate delivery to diverse cell types, and exploring different micellar formulations for optimizing therapeutic efficacy against various bacterial infections.

## Figures and Tables

**Table 1:** The physicochemical characteristics of amphiphilic conjugates derived from chitosan and heparin, with oleic or lipoic acid residues attached. The parameters of micellar solutions in a phosphate-buffered saline solution (0.01 M, pH 7.4) at a temperature of 37 °C are given. Data represent the mean ± standard deviation (SD), calculated from 3 to 5 independent replicates. Confidence intervals were determined using Student’s *t*-test.

Designation *	Chemical Formula **	Polymer Modification Degree per Glycoside Unit, %	Hydrodynamic Diameter, nm ***	ζ-Potential, mV ****
M1	Chit5-LA	25 ± 3	180 ± 40	+12 ± 2
M2	Chit5-OA	22 ± 4	250 ± 60	+15 ± 1
M3	Hep-OA	13 ± 2	160 ± 20	–7 ± 1
M4	Hep-LA	10 ± 2	125 ± 10	–10 ± 2

* M stands for micellar, ** Chit5—chitosan 5 kDa, Hep—heparin 12–14 kDa, LA—lipoic acid residue, OA—oleic acid residue. *** determined using AFM. **** determined using DLS.

**Table 2:** The maximal degree of incorporation of substances into micelles is expressed in mass percentages. Data represent the mean ± standard deviation (SD), calculated from 3 to 5 independent replicates. Confidence intervals were determined using Student’s *t*-test.

Micelle Type/Drug	Curcumin 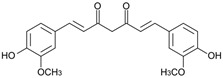	MUmb 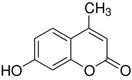	AMC 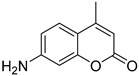
Chit5-LA (M1)	6.2 ± 0.4	17 ± 2	13 ± 1
Chit5-OA (M2)	13 ± 1	18 ± 2	14 ± 2
Hep-OA (M3)	10 ± 1	20 ± 1	21 ± 1
Hep-LA (M4)	3.5 ± 0.5	18 ± 2	20 ± 2

**Table 3:** The values of the overlap integrals J(λ) and Förster radii R_0_ for RFP-drug pairs.

FRET Parameters with RFP	J(λ), nm^4^/(M × cm)	R_0_, Å
Curcumin	2.50 × 10^15^	28
Curcumin in Chit-OA	1.64 × 10^15^	43
AMC	9.30 × 10^13^	33
AMC in Chit-OA	9.30 × 10^13^	33
MUmb	1.32 × 10^14^	35
MUmb in Chit-OA	1.37 × 10^14^	36

**Table 4:** Physico-chemical parameters of the inclusion of MUmb in polymeric micelles. PBS (0.01 M, pH 7.4). T = 37 °C. Data represent the mean ± standard deviation (SD), calculated from 3 to 5 independent replicates. Confidence intervals were determined using Student’s *t*-test.

Micelles	Chit-LA	Chit-OA	Hep-OA	Hep-LA
–lg *K*_A_	3.16 ± 0.09	3.09 ± 0.30	5.65 ± 0.26	6.71 ± 0.42
–lg *K*_d_	3.75 ± 0.19	3.79 ± 0.65	4.69 ± 0.43	4.97 ± 0.62
n	0.84 ± 0.02	0.81 ± 0.06	1.20 ± 0.05	1.35 ± 0.08
R-square	0.998	0.984	0.994	0.989

**Table 5:** RFP fluorescence intensity in a Petri dish experiment using bacteria expressing red fluorescent protein (RFP) as a visual platform for assessing the permeability of 0.1 mg/mL formulations of AMC, MUmb, and curcumin, both free and encapsulated in micelles. For curcumin, a DMSO sample with a concentration of 1 mg/mL was also used as a positive control. The negative control was PBS (0.01 M, pH 7.4). T = 37 °C. Data represent the mean ± standard deviation (SD), calculated from 3 to 5 independent replicates. Confidence intervals were determined using Student’s *t*-test.

RFP Fluorescence Intensity, a.u.			
Form/Compound	MUmb	AMC	Curcumin
In PBS	180 ± 11	174 ± 8	132 ± 4 (in PBS)/178 ± 10 (in DMSO)
In Chit5-LA (M1)	226 ± 8	133 ± 4	135 ± 5
In Chit5-OA (M2)	208 ± 10	146 ± 7	148 ± 7
In Hep-OA (M3)	188 ± 5	205 ± 11	149 ± 5
In Hep-LA (M4)	195 ± 13	228 ± 15	144 ± 6
Control (RFP only)	183 ± 7	124 ± 3	125 ± 4

**Table 6:** The effect of different drug formulations on *E. coli* cells viability (%). The initial values were 2 × 10^7^ CFU/mL. T = 37 °C. Data represent the mean ± standard deviation (SD), calculated from 3 to 5 independent replicates. Confidence intervals were determined using Student’s *t*-test. The *p*-values for micellar formulation compared to non-micellar drugs were < 0.01.

Sample 0.1 mg/mL	Cell Viability, %		
	Curcumin	MUmb	AMC
in PBS	66 ± 3	55 ± 4	60 ± 4
in Chit5-LA (M1)	39 ± 2	32 ± 1	47 ± 5
in Chit5-OA (M2)	41 ± 4	25 ± 3	42 ± 3
in Hep-OA (M3)	24 ± 1	30 ± 2	36 ± 2
in Hep-LA (M4)	36 ± 2	41 ± 4	40 ± 3
in DMSO (1 mg/mL) positive control	52 ± 1	27 ± 2	35 ± 4

## Data Availability

The data presented in this study are available in the main text and Supplementary Materials.

## Supplementary Materials

The following supporting information can be downloaded at: https://www.mdpi.com/article/10.3390/polym17060790/s1, Figure S1. The schemes of synthesis of amphiphilic polymers: Chit5-LA, Chit5-OA, Hep-OA and Hep-LA. Figure S2. The results of fluorescence pyrene probe analysis to determine the critical micelle concentration (CMC) for chitosan-LA (Chit-LA), chitosan-OA (Chit-OA), heparin-OA (Hep-OA), and heparin-LA (Hep-LA). The normalized fluorescence of pyrene excimer is plotted against the (logarithm of polymer concentration, expressed in µg per mL). The point at which 50% of fluorescence is achieved corresponds to the formation of micelles. The experiments were conducted using PBS buffer (0.01 M, pH 7.4) at a temperature of 37 °C and an excitation wavelength of 340 nm. Table S1. Pharmacokinetic parameters of Dox in free and micellar form in Wistar rats. From our previous work [10.3390/polym16152132].
