# Supplementary material for: Targeted Delivery Inside the Cells Directly Visualized with Förster Resonance Energy Transfer (FRET)
Source: Polymers (Basel). 2025 Mar 16;17(6):790. doi: 10.3390/polym17060790 (PMC11944702; doi:10.3390/polym17060790)
Supplement: Supplementary file 1 [file polymers-17-00790-s001.zip › polymers-3493901-supplementary.pdf]

Article

Targeted Delivery Inside the Cells Directly Visualized with Förster resonance energy transfer (FRET)

Igor D. Zlotnikov, Natalya G. Belogurova and Elena V. Kudryashova *

Faculty of Chemistry, Lomonosov Moscow State University, Leninskie Gory, 1/3, 119991 Moscow, Russia; zlotnikovid@my.msu.ru, nbelog@mail.ru

* Correspondence: helenakoudriachova@yandex.ru

Figure S1. The schemes of synthesis of amphiphilic polymers: Chit5-LA, Chit5-OA, Hep-OA, and Hep-LA.

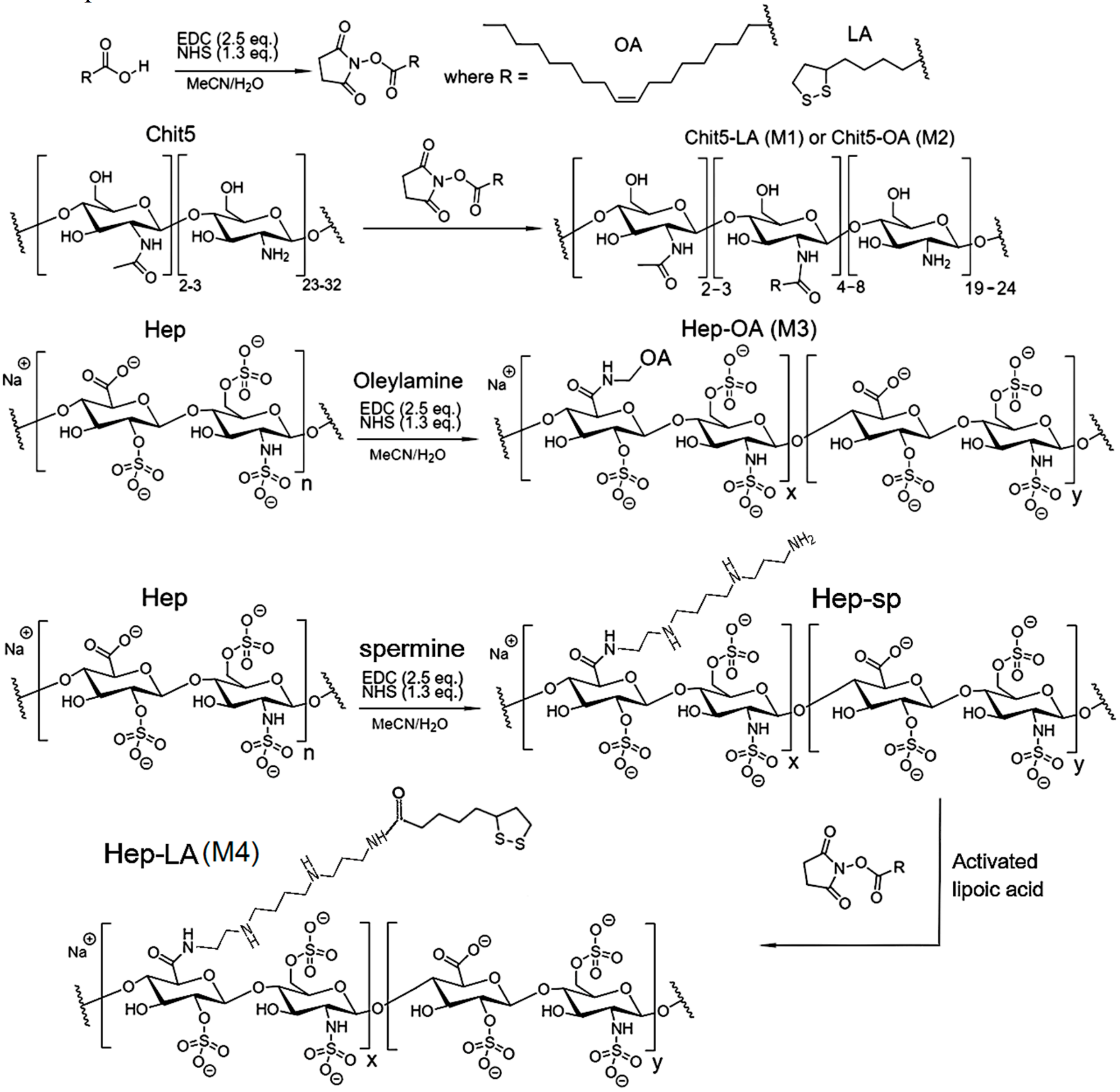

Figure S2. The results of fluorescence pyrene probe analysis to determine the critical micelle concentration (CMC) for chitosan-LA (Chit-LA), chitosan-OA (Chit-OA), heparin-OA (Hep-OA), and heparin-LA (Hep-LA). The normalized fluorescence of pyrene excimer is plotted against the (logarithm of polymer concentration, expressed in µg per mL). The point at which 50% of fluorescence is achieved corresponds to the formation of micelles. The experiments were conducted using PBS buffer (0.01 M, pH 7.4) at a temperature of 37°C and an excitation wavelength of 340 nm.

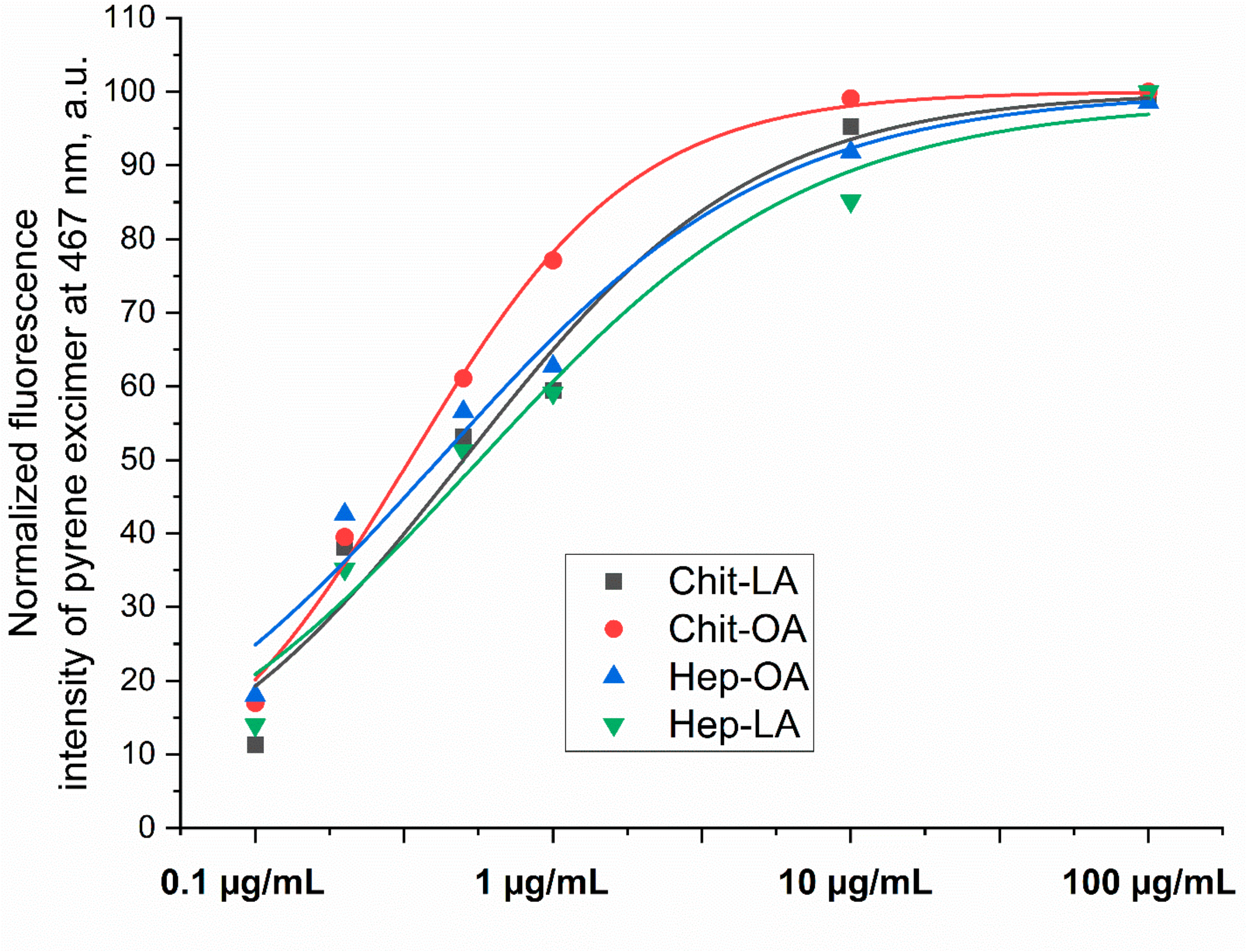

Table S1. Pharmacokinetic parameters of Dox in free and micellar form in Wistar rats. From our previous work [10.3390/polym16152132].

Dox Formulation	Half-Elimination Time, min	Area under Curve 0–480 Min (~Effective Concentration)
**Dox free**	220	800
**DoxM1**	>500	960
**DoxM2**	250	1270
